# Feasibility of Using a Single Heart Rate–Based Measure for Real-time Feedback in a Voluntary Deep Breathing App for Children: Data Collection and Algorithm Development

**DOI:** 10.2196/16639

**Published:** 2020-09-23

**Authors:** Christian L Petersen, Matthias Görges, Evgenia Todorova, Nicholas C West, Theresa Newlove, J Mark Ansermino

**Affiliations:** 1 Department of Anesthesiology, Pharmacology & Therapeutics The University of British Columbia Vancouver, BC Canada; 2 Research Institute BC Children’s Hospital Vancouver, BC Canada; 3 Department of Psychology The University of British Columbia Vancouver, BC Canada; 4 Department of Psychology BC Children’s Hospital Vancouver, BC Canada

**Keywords:** pediatric pain, respiratory sinus arrhythmia, biofeedback, pulse oximetry, mobile health, anxiety, diaphragmatic breathing, self-regulation

## Abstract

**Background:**

Deep diaphragmatic breathing, also called belly breathing, is a popular behavioral intervention that helps children cope with anxiety, stress, and their experience of pain. Combining physiological monitoring with accessible mobile technology can motivate children to comply with this intervention through biofeedback and gaming. These innovative technologies have the potential to improve patient experience and compliance with strategies that reduce anxiety, change the experience of pain, and enhance self-regulation during distressing medical procedures.

**Objective:**

The aim of this paper was to describe a simple biofeedback method for quantifying breathing compliance in a mobile smartphone app.

**Methods:**

A smartphone app was developed that combined pulse oximetry with an animated protocol for paced deep breathing. We collected photoplethysmogram data during spontaneous and subsequently paced deep breathing in children. Two measures, synchronized respiratory sinus arrhythmia (RSA_sync_) and the corresponding relative synchronized inspiration/expiration heart rate ratio (HR-I:E_sync_), were extracted from the photoplethysmogram.

**Results:**

Data collected from 80 children aged 5-17 years showed a positive RSA_sync_ effect in all participants during paced deep breathing, with a median (IQR; range) HR-I:E_sync_ ratio of 1.26 (1.16-1.35; 1.01-1.60) during paced deep breathing compared to 0.98 (0.96-1.02; 0.82-1.18) during spontaneous breathing (median difference 0.25, 95% CI 0.23-0.30; *P*<.001). The measured HR-I:E_sync_ values appeared to be independent of age.

**Conclusions:**

An HR-I:E_sync_ level of 1.1 was identified as an age-independent threshold for programming the breathing pattern for optimal compliance in biofeedback.

## Introduction

As a child’s psychological well-being during hospital visits is associated with improved health outcomes, it is imperative to find ways to minimize the stress and anxiety that children experience during medical procedures [1]. Studies have shown that decreased anxiety is associated with not only decreased distress but also decreased pain and less negative attitudes toward future medical procedures [2]. Therefore, providing developmentally appropriate strategies to support children’s coping before, during, and after medical procedures should be considered an important element of care.

Deep diaphragmatic breathing (sometimes referred to as belly breathing in pediatric settings) typically produces a relaxed state and is considered a behavioral coping strategy to reduce anxiety in children undergoing medical procedures [3]. Teaching deep diaphragmatic breathing is a popular behavioral intervention used by health care professionals, which affects both the physiological and psychological outcomes of patients. This technique has been found to ease procedural distress in children with cystic fibrosis [4] and anxiety in children with asthma [5]. In addition to the psychophysiological benefits of deep diaphragmatic breathing, intermittent periods of a slow respiratory rate (in the range of 6 breaths/minute) can have a direct positive impact on the cardiorespiratory health of patients. They increase the resting oxygen saturation [6] and baroreflex sensitivity [7-9] while reducing chemoreflex actuation [10] and muscle nerve sympathetic activity [11].

Compliance with breathing protocols is optimized when children are taught through instruction, modelling, and in-vivo coaching by a health care provider typically assigned to support the child during a medical procedure. Children who have previously been identified as having significant difficulties in participating in medical procedures are often referred to Child Life specialists or other providers assigned to support children; however, many children who experience distress during medical procedures are not recommended interventions and do not have the opportunity to access coping strategies. In the absence of this active teaching and coaching to belly breathe during a procedure, children are often unable to belly breathe successfully, instead focusing on the procedure that is the source of distress and discomfort. As children are increasingly surrounded by technology, both at home and in educational settings, teaching deep breathing through a smartphone app can increase accessibility to coping strategies for children undergoing medical procedures. The smartphone app acts as a biofeedback game that simultaneously enables children to successfully engage in belly breathing while also providing active distraction that can further help reduce the experience of pain [12].

There is limited research published on using biofeedback apps to teach relaxation to children in clinical settings; however, evidence supports the effectiveness of biofeedback as an intervention for invasive procedures in children with cancer [13], in patients post cardiac surgery [14], and in children with asthma [15]. A systematic review of apps for the management of pain and stress, including 11 breathing-related apps, cautioned that a majority were developed by lay-professionals, were intended to be used by adults, and had not been formally evaluated [16]. Furthermore, few of these apps used sensors for feedback; those that did, used the phone’s accelerometer placed on the xiphoid process or, more recently, obtained the heart rate from an Apple Watch [16].

Respiratory sinus arrhythmia refers to the heart rate variation that occurs during the respiratory cycle, by which the heart rate increases during inspiration and decreases during expiration [17,18]. This effect is especially pronounced in children [19] and decreases with age and declining cardiovascular health [20]. The change in rate is due to respiratory-induced changes in intrathoracic pressure. The change in pressure leads to changes in cardiac output that lead to a reflex-mediated change in heart rate. Deep diaphragmatic breathing enhances the respiratory sinus arrhythmia amplitude, as activation of pulmonary stretch receptors increases pulmonary vagal inhibition. The photoplethysmogram (PPG) waveform, obtained from a pulse oximeter, contains detailed information about heart rate variability. Although heart rate variability is traditionally extracted from an electrocardiogram, the PPG can be used to extract equivalent measures [21]. It is therefore plausible that PPG-derived respiratory sinus arrhythmia could be the basis of a deep breathing biofeedback system for children.

This study uses a previously developed smartphone audio-based pulse oximeter [22] as a biofeedback measure for a smartphone game that teaches and promotes deep diaphragmatic breathing in children. The aim of this paper was to describe the creation of a simple indicator to quantify compliance with deep breathing patterns, which can be used in the fully programmed biofeedback app.

## Methods

### Study Design

We prospectively collected PPG data from a cohort of volunteer child participants during sequential spontaneous breathing and paced deep breathing, guided by a smartphone app, in order to establish an appropriate measure that can be implemented for future use in real–time smartphone-based biofeedback.

### Ethical Approval

The study was approved by the University of British Columbia/Children's and Women's Health Centre of British Columbia Research Ethics Board (H14-02577). All procedures performed in studies involving human participants were in accordance with the ethical standards of the institutional and/or national research committee and with the 1964 Helsinki declaration and its later amendments or comparable ethical standards.

Informed parental consent and child assent was obtained from all individual participants included in the study.

### Equipment

A smartphone app for iOS and Android was developed using the cross-platform open source LambdaNative framework [23,24]. In an effort to engage children, the app features a happy protagonist, named Johnny Bellybreath, in a hot air balloon [25], who inhales and exhales bubbles. As he inhales, animated bubbles enter his nose. As he exhales, the bubbles reappear from his mouth; during the pause between inhalation and exhalation, there are no bubbles shown on the screen. The goals of the biofeedback game are as follows: (1) to teach a voluntary deep breathing protocol; (2) to detect compliance to the breathing protocol using an attached audio-based pulse oximeter sensor; (3) to raise the hot air balloon as the child belly breathes successfully, as determined by the relative synchronized expiration to inspiration heart rate ratio (see Data Analysis section); and (4) to reinforce continued belly breathing by keeping the balloon rising while exponentially increasing altitude and increasing the “altitude score” of the game, reaching 1,000,000 m after approximately 2 minutes. In the final application, the scenery changes as the balloon rises driven by biofeedback, until it eventually reaches outer space.

**Figure 1:**
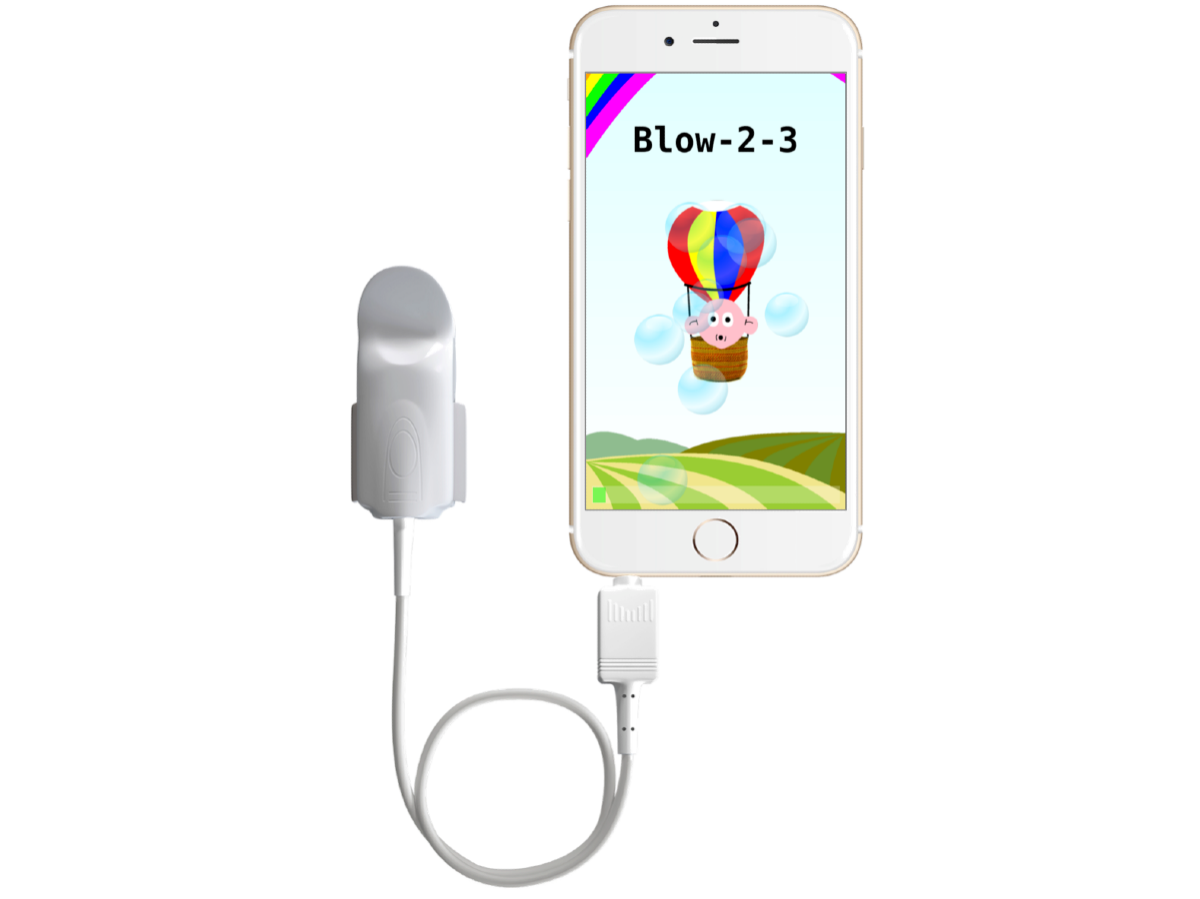
Pulse oximetry sensor– and smartphone–based animated deep breathing trainer. The animated character inhales bubbles through the nose and exhales them through the mouth at 6 breaths per minute.

For the purpose of data collection, the smartphone app contains two nonfeedback modes of operation: Blank mode and Training mode. In Blank mode, the screen is blank, and the app simply measures and records the PPG using the pulse oximeter sensor. In Training mode, the app displays a stationary hot air balloon (no biofeedback), and the character blows bubbles in accordance with the slow breathing protocol at 6 breaths per minute (3 seconds inhale, 3 seconds hold, 3 seconds exhale, and 1 second pause/transition) while PPG data is recorded. The Training mode also shows the 3-step breathing instructions on the screen as “Breathe-2-3,” “Hold-2-3,” and “Blow-2-3” (Figure 1).

### Participants and Data Collection

A kiosk was set up in the hallways of the British Columbia Children’s Hospital to recruit participants. The smartphone app was installed on an iPad (Apple Inc) and mounted on a stand adjusted to be at eye level for a seated child. Inclusion criteria for children to participate in the study were (1) age, 5-17 years; (2) ability to speak English; (3) no significant developmental or intellectual disability; and (4) no severe cardiovascular or respiratory condition that could either inhibit their ability to perform deep breathing or significantly affect their heart rate variability.

Participants were seated in a chair in front of the iPad with the audio pulse oximeter placed on an index finger. During spontaneous breathing, the PPG was recorded for 1 minute using the Blank mode of the smartphone application; participants did not receive specific instructions, as the purpose was to emulate random breathing when children were not paying attention to the game. After collecting spontaneous data, the deep diaphragmatic breathing technique was explained to the participants and the Training mode of the application was demonstrated by a hospital volunteer using a script and accompanying pictures. Then, the smartphone app was put into Training mode and the PPG was collected for 2 minutes of paced deep breathing. The phase of the deep breathing cycle was fixed for all collected data sets, allowing a systematic comparison of the heart rate between participants at different phases of the breathing cycle.

### Data Analysis

Heart rates were extracted from the PPG signal on a beat-to-beat basis to quantify the variability of respiratory sinus arrhythmia in children. A 1-second window of heart rate was recorded 2.5-3.5 seconds (typically the end of the inspiratory cycle in spontaneously breathing children) into the breathing cycle for inspiration, and a second 1-second window of heart rate at 7.5-8.5 seconds for expiration (typically the middle of the expiratory cycle). The timing of 1-second windows corresponded to the timing of the animated pacing bubbles in the game and, when successfully playing the game, the children should have been in inspiration/expiration at those moments. The width of the window was a compromise, rewarding compliance to the animation while at the same time allowing for natural variations in each breathing cycle. Finally, during spontaneous breathing, the animation was not shown, but the heart rate window timing was identical to that used during paced deep breathing.

**Figure 2:**
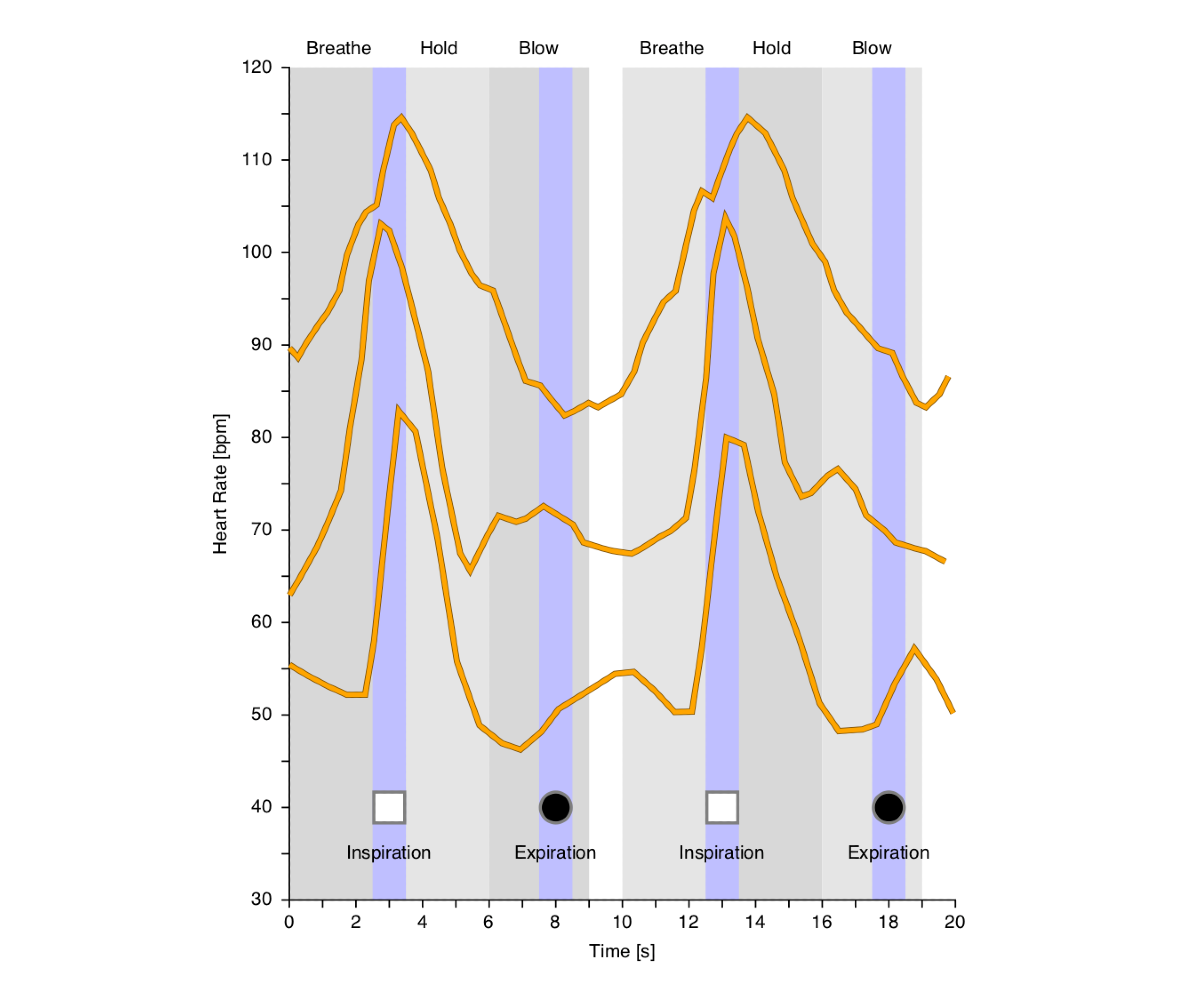
Example tachograms from 3 separate participants for two consecutive breathing cycles, illustrating interpatient variance in respiratory sinus arrhythmia slope and maxima/minima. The signal is sampled in two 1-second windows (highlighted in blue), during inspiration (white) and expiration (black) respectively, to extract the synchronized respiratory sinus arrhythmia measure.

The heart beats were extracted using a standard peak detection method; specifically, using zero-crossings in the difference signal after band-limiting it to 0.5-4Hz using a fourth-order filter (Figure 2). This sampling gives an equitemporal distribution of the inspiration/expiration updates (5 seconds), which is convenient for the real–time feedback implementation. The difference between the average heart rates (HR) recorded during HR_inspiration_ and HR_expiration_, was used to determine a synchronized respiratory sinus arrhythmia measure, RSA_sync_ = HR_inspiration_ - HR_expiration_, and the corresponding relative synchronized expiration/inspiration ratio, HR-I:E_sync_ = HR_inspiration_/HR_expiration_.

For each participant, the average HR-I:E_sync_ ratio was obtained. Results were grouped according to the participants’ ages: 5-7 years, 8-10 years, 11-13 years, and 14-17 years. The median and IQR were also determined. Finally, HR-I:E_sync_ ratios for all participants were compared using the Wilcoxon signed-rank test with the 95% confidence interval of the median difference to identify the nonoverlapping area between groups, to be used as a threshold for the breathing pattern performance.

## Results

A total of 109 children entered the study over the course of 53 days. Of these, 16 participants did not complete the study, 9 participants were excluded because of respiratory conditions, and 4 met other exclusion criteria. The remaining 80 eligible participants (34 boys, 46 girls) were separated into 4 age groups: 5-7 years (n=18), 8-10 years (n=20), 11-13 years (n=21), and 14-17 years (n=21) (Table 1).

**Table 1:** Age groups characteristics. Data are reported as median (IQR).

Age group (years)	Age (years)	Participants (N=80), n	
		Female (n=46)	Male (n=34)
5-7	7 (6-7)	12	6
8-10	9 (8-10)	12	8
11-13	11 (11-13)	7	14
14-17	15 (15-16)	15	6

**Figure 3:**
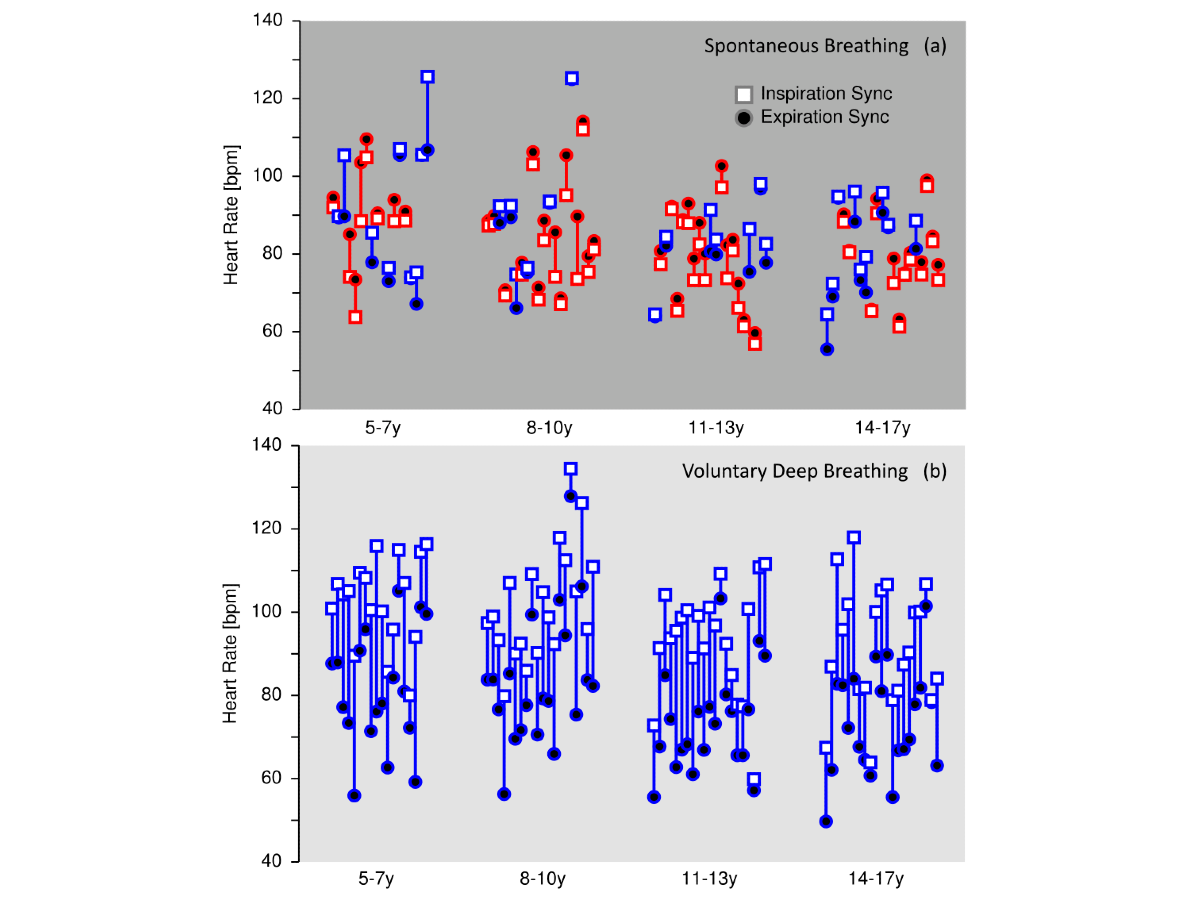
Measured heart rates at inspiration (white square bullets) and expiration (black round bullets) using (a) spontaneous breathing (Blank mode), and (b) the paced deep breathing at 6 breaths per minute (Training mode). Positive response is indicated in blue color, and negative response is indicated in red color.

During the spontaneous breathing (Blank mode), there appeared to be no systematic difference between the inspiration and expiration synchronization, as the participants’ natural breathing patterns did not synchronize with the paced breathing pattern (Figure 3a). On the other hand, during paced deep breathing (Training mode), all participants exhibited a higher heart rate at inspiration than at expiration, equivalent to a positive RSA_sync_ response (Figure 3b).

All 80 participants exhibited a positive RSA_sync_ value in the Training mode of the app, with a median 26% increase in heart rate at inspiration over expiration, compared to a 2% decrease in the Blank mode of the app (Table 2). The timing of the peak heart rates at inspiration was well determined across participants and located at the end of the inspiratory segment of the breathing cycle. The minimum heart rate at expiration was dependent on the individual dynamic inter-breath heart rate variations, but generally located in the middle of the expiratory segment of the cycle.

**Table 2:** Heart rates observed at inspiration and expiration, synchronized respiratory sinus arrhythmia measure (RSA_sync_), and relative synchronized expiration/inspiration ratio (HR-I:E_sync_), split by age groups and feedback mode (spontaneous and deep breathing). Data are reported as median (IQR).

Age group (years)	HR^a^ inspiration (bpm^b^)		HR expiration (bpm)		RSA_sync_ (bpm)		HR-I:E_sync_	
	Spontaneous	Deep breathing	Spontaneous	Deep breathing	Spontaneous	Deep breathing	Spontaneous	Deep breathing
5-7	82.5 (73.3-88)	95.5 (89.0-100.8)	80.7 (75.4-88.0)	73.2 (65.6-77.3)	–2.8 (–5.5 to 1.2)	22.0 (12.2-24.1)	0.97 (0.95-1.01)	1.30 (1.19-1.35)
8-10	79.2 (73.3-88.6)	90.3 (81.5-101.9)	80.3 (73.3-88.3)	72.2 (64.5-82.5)	–0.4 (–1.9 to 3.3)	18.3 (13.9-23.3)	0.99 (0.98-1.05)	1.28 (1.19-1.36)
11-13	88.6 (76.4-104.9)	105.1 (95.8-109.4)	90.5 (77.9-103.6)	81.0 (72.2-90.8)	0.0 (–4.7 to 3.4)	18.8 (13.2-29.1)	1.00 (0.96-1.05)	1.21 (1.14-1.41)
14-17	82.4 (74.6-92.7)	98.9 (92.4-109.6)	88.3 (77.2-90.6)	80.8 (74.5-87.5)	–2.1 (–3.4 to 0.3)	19.8 (14.6-22.2)	0.98 (0.95-1.00)	1.24 (1.16-1.30)

^a^HR: heart rate

^b^bpm: beats per minute

**Figure 4:**
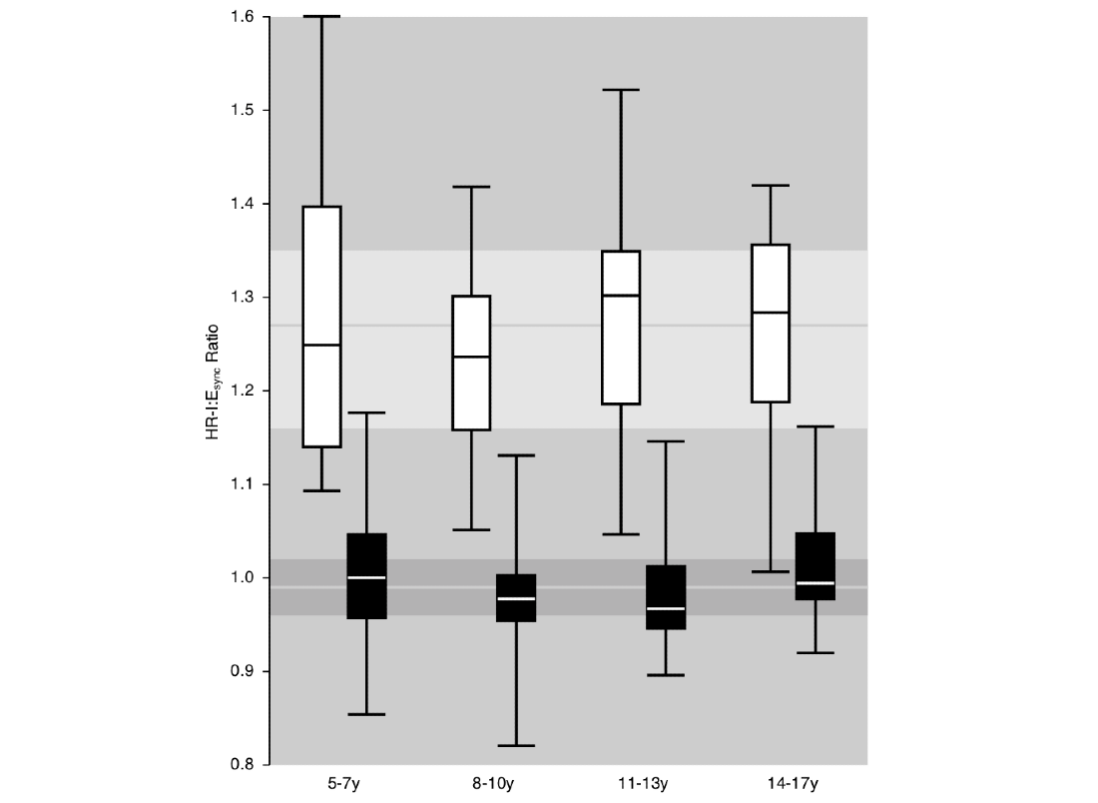
Age group–specific median synchronized inspiration/expiration heart rate ratios during paced deep breathing (Training mode; white) and spontaneous breathing (Blank mode; black). The horizontal bands indicate the total first to third quartile for the two data sets, with the median indicated using a horizontal line.

The median (IQR; range) HR-I:E_sync_ across the entire population was 1.26 (1.16-1.35; 1.01-1.60) during paced deep breathing and 0.98 (0.96-1.02; 0.82-1.18) during spontaneous breathing (Figure 4); the median difference was 0.26 with a 95% CI of 0.23-0.30 (*P*<.001).

The measured HR-I:E_sync_ values appeared to be age independent (Figure 4). Hence, a single HR-I:E_sync_ threshold of 1.1 was selected as a reasonable threshold for breathing compliance during biofeedback.

## Discussion

In this study, we observed a pronounced RSA_sync_ during paced deep breathing and lack of synchronization during spontaneous breathing. The synchronized differences were sufficiently distinct to identify a single heart rate–based measure (HR-I:E_sync_ = 1.1) for biofeedback in all participants. This HR-I:E_sync_ threshold can be universally applied to children of all ages, despite the large inherent interpatient variability in this demographic. It is unexpected that the RSA_sync_ magnitude is relatively constant between age groups, as it is known that respiratory sinus arrhythmia naturally decreases with age. Instead, results show a moderate reversal, with a slightly lower effect in the youngest age group and an elevated effect in the oldest age group. This could be due to differences in compliance, increased respiratory effort by older children, or the smaller data sets in the two limiting age groups.

The fact that the RSA_sync_ effect is pronounced even in the youngest age group demonstrates that the visual breathing instructions in the Training mode of the app is an effective way of pacing deep breathing in small children. It suggests that the smartphone biofeedback app could be used in a wide age range of children, possibly even in children younger than the participants recruited in this study.

The particular shape of the respiratory sinus arrhythmia response was found to be different between participants, without any clear age dependence or other systematic relation. Some participants exhibited a broad sawtooth-like response in heart rate during the cycle, while others showed a narrow response characterized by a rapid increase and decrease of heart rate at inspiration and a reproducible substructure in between breaths. It is unclear whether this difference is due to physiological differences between participants, individual variations in interpretation of the breathing protocol, or a combination thereof.

The conventional method for extracting respiratory sinus arrhythmia from the heart rate tachogram involves resampling and performing a Fourier transformation on a long temporal window. However, this frequency domain method does not suit the purposes of biofeedback, as a real–time response that can be updated during each breathing segment is required. The proposed RSA_sync_ measure underestimates true respiratory sinus arrhythmia, as the true heart rate extrema may fall outside of the sampling windows. However, the advantage of using RSA_sync_ for biofeedback is that spontaneous breathing should yield a net RSA_sync_ of zero due to the lack of synchronization, while compliant deep diaphragmatic breathing is expected to yield a value of RSA_sync_ approaching the full arrhythmia effect.

### Limitations

This study presents preliminary data. The biofeedback process and threshold selected for belly breathing protocol compliance feedback were not evaluated as part of this study. The children involved in this study were volunteers not undergoing any medical procedure while they were participating in the study. Further research is required to evaluate whether these results will translate to children experiencing the pain and/or stress of a medical condition or procedure, which paced deep breathing (belly breathing) is intended to alleviate.

### Conclusions

The use of PPG as biofeedback for deep breathing in children was investigated. Our results suggest that this is a feasible approach and that a single universal threshold in HR-I:E_sync_ ratio of 1.1 will be sufficient to encode real–time feedback in the app for all ages evaluated.

The next phases of the study involve programming the smartphone biofeedback in accordance to these findings, and comparing the app to traditional teaching methods by measuring the respiratory sinus arrythmia magnitude in both groups to determine its effectiveness in reinforcing effective deep diaphragmatic breathing in children. Subsequently, the app will be investigated as a tool to improve self-regulation and coping and to minimize pre-procedural anxiety and the experience of pain in children undergoing medical procedures. The fully programmed biofeedback deep breathing application represents a new tool that can encourage children to engage in a simple behavioral intervention with the potential to reduce the experience of pain and anxiety of medical procedures during hospital visits and in other settings. It may provide children with an opportunity to engage in a proactive, child-friendly activity, giving them a sense of control during painful, but necessary, medical procedures. This is important, as children who experience distress even during routine medical procedures can carry this negative experience into subsequent health care interactions. Thus, this tool may provide children with a coping strategy that could improve their health care journey.

## Abbreviations

HR: heart rate
HR-I:E_sync_: synchronized inspiration/expiration heart rate ratio
PPG: photoplethysmogram
RSA_sync_: synchronized respiratory sinus arrhythmia
